# *N*′-[1-(2-Hy­droxy­phen­yl)ethyl­idene]-2-meth­oxy­benzohydrazide

**DOI:** 10.1107/S1600536810020477

**Published:** 2010-06-05

**Authors:** Si-Yu Yue, Jiu-Fu Lu

**Affiliations:** a School of Chemistry and Environmental Science, Shaanxi University of Technology, Hanzhong 723000, People’s Republic of China

## Abstract

There are two independent mol­ecules in the asymmetric unit of the title compound, C_16_H_16_N_2_O_3_, in which the dihedral angles between the two aromatic rings are 13.0 (3) and 6.4 (3)°. Intra­molecular O—H⋯N and N—H⋯O hydrogen bonds are observed in both mol­ecules, forming *S*(6) rings in all cases.

## Related literature

For related structures, see: Lu *et al.* (2008*a* ▶,*b* ▶,*c* ▶); Xiao & Wei (2009 ▶); He (2008 ▶); Shi *et al.* (2007 ▶). For reference bond-length data, see: Allen *et al.* (1987 ▶).
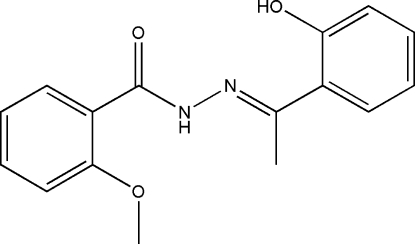


## Experimental

### 

#### Crystal data

C_16_H_16_N_2_O_3_
*M* *_r_* = 284.31
Monoclinic, 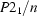

*a* = 11.5610 (12) Å
*b* = 10.8074 (11) Å
*c* = 22.544 (2) Å
β = 92.244 (5)°
*V* = 2814.6 (5) Å^3^
*Z* = 8
Mo *K*α radiation
μ = 0.09 mm^−1^
*T* = 298 K
0.20 × 0.17 × 0.17 mm

#### Data collection

Bruker APEXII CCD area-detector diffractometer
Absorption correction: multi-scan (*SADABS*; Sheldrick, 2004 ▶) *T*_min_ = 0.981, *T*_max_ = 0.984
16351 measured reflections
6065 independent reflections
3390 reflections with *I* > 2σ(*I*)
*R*_int_ = 0.032

#### Refinement

*R*[*F* ^2^ > 2σ(*F* ^2^)] = 0.050
*wR*(*F* ^2^) = 0.146
*S* = 1.01
6065 reflections
391 parameters
2 restraints
H atoms treated by a mixture of independent and constrained refinement
Δρ_max_ = 0.18 e Å^−3^
Δρ_min_ = −0.22 e Å^−3^

### 

Data collection: *APEX2* (Bruker, 2004 ▶); cell refinement: *SAINT* (Bruker, 2004 ▶); data reduction: *SAINT*; program(s) used to solve structure: *SHELXS97* (Sheldrick, 2008 ▶); program(s) used to refine structure: *SHELXL97* (Sheldrick, 2008 ▶); molecular graphics: *SHELXTL* (Sheldrick, 2008 ▶); software used to prepare material for publication: *SHELXTL*.

## Supplementary Material

Crystal structure: contains datablocks global, I. DOI: 10.1107/S1600536810020477/wn2390sup1.cif

Structure factors: contains datablocks I. DOI: 10.1107/S1600536810020477/wn2390Isup2.hkl

Additional supplementary materials:  crystallographic information; 3D view; checkCIF report

## Figures and Tables

**Table 1:** Hydrogen-bond geometry (Å, °)

*D*—H⋯*A*	*D*—H	H⋯*A*	*D*⋯*A*	*D*—H⋯*A*
O1—H1⋯N1	0.82	1.82	2.535 (2)	144
O4—H4⋯N3	0.82	1.82	2.541 (2)	145
N2—H2⋯O3	0.90 (1)	1.83 (2)	2.591 (2)	141 (2)
N4—H4*B*⋯O6	0.90 (1)	1.88 (2)	2.619 (2)	138 (2)

**Fig. 1.**
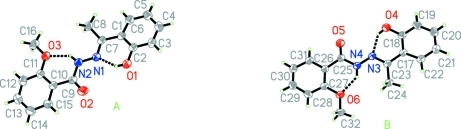
The molecular structure of the asymmetric unit of the title compound, showing 30% probability displacement ellipsoids and the atom-numbering scheme. The dashed lines indicate hydrogen bonds.

## supplementary crystallographic information

### Comment

Recently we have reported a number of Schiff bases derived from the condensation of aldehydes or ketones with benzohydrazides (Lu *et al.*, 2008*a*,b,c) We report here the crystal structure of the new title Schiff base compound.

In the crystal structure of the title compound, Fig. 1, there are two independent molecules in the asymmetric unit. The bond lengths have normal values (Allen *et al.*, 1987), and are comparable to those observed in similar compounds (Xiao & Wei, 2009; He, 2008; Shi *et al.*, 2007). The dihedral angles between the two aromatic rings in molecules A and B are 13.0 (3) and 6.4 (3)°, respectively. Intramolecular O—H···N and N—H···O hydrogen bonds are observed in the molecules (Table 1).

### Experimental

The title compound was prepared by the Schiff base condensation of 1-(2-hydroxyphenyl)ethanone (0.1 mol, 13.6 g) and 2-methoxybenzohydrazide (0.1 mol, 16.6 g) in 95% ethanol (70 ml). The excess ethanol was removed by distillation. The resulting colourless solid was filtered and washed with ethanol. Single crystals suitable for X-ray diffraction were obtained by slow evaporation of a 95% ethanol solution at room temperature.

### Refinement

H2 attached to N2, and H4B attached to N4 were located in a difference map and refined with the N—H distance restrained to 0.90 (1) Å. The other H atoms were positioned geometrically (C—H = 0.93–0.96 Å and O—H = 0.82 Å) and refined using a riding model, with with *U*_iso_(H) = x*U*_eq_(attached atom), where x = 1.5 for methyl H and hydroxyl H, 1.2 for all other carbon-bound H atoms. A rotating group model was used for the methyl and hydroxyl groups.

### Crystal data

**Table:**

C_16_H_16_N_2_O_3_	*F*(000) = 1200
*M**_r_* = 284.31	*D*_x_ = 1.342 Mg m^−^^3^
Monoclinic, *P*2_1_/*n*	Mo *K*α radiation, λ = 0.71073 Å
*a* = 11.5610 (12) Å	Cell parameters from 3247 reflections
*b* = 10.8074 (11) Å	θ = 2.5–24.5°
*c* = 22.544 (2) Å	µ = 0.09 mm^−^^1^
β = 92.244 (5)°	*T* = 298 K
*V* = 2814.6 (5) Å^3^	Block, colourless
*Z* = 8	0.20 × 0.17 × 0.17 mm

### Data collection

**Table:**

Bruker APEXII CCD area-detector diffractometer	6065 independent reflections
Radiation source: fine-focus sealed tube	3390 reflections with *I* > 2σ(*I*)
graphite	*R*_int_ = 0.032
ω scans	θ_max_ = 27.0°, θ_min_ = 1.8°
Absorption correction: multi-scan (*SADABS*; Sheldrick, 2004)	*h* = −14→14
*T*_min_ = 0.981, *T*_max_ = 0.984	*k* = −13→13
16351 measured reflections	*l* = −28→28

### Refinement

**Table:**

Refinement on *F*^2^	Primary atom site location: structure-invariant direct methods
Least-squares matrix: full	Secondary atom site location: difference Fourier map
*R*[*F*^2^ > 2σ(*F*^2^)] = 0.050	Hydrogen site location: inferred from neighbouring sites
*wR*(*F*^2^) = 0.146	H atoms treated by a mixture of independent and constrained refinement
*S* = 1.01	*w* = 1/[σ^2^(*F*_o_^2^) + (0.0661*P*)^2^ + 0.2197*P*] where *P* = (*F*_o_^2^ + 2*F*_c_^2^)/3
6065 reflections	(Δ/σ)_max_ < 0.001
391 parameters	Δρ_max_ = 0.18 e Å^−^^3^
2 restraints	Δρ_min_ = −0.22 e Å^−^^3^

### Special details

**Table:**

Geometry. All e.s.d.'s (except the e.s.d. in the dihedral angle between two l.s. planes) are estimated using the full covariance matrix. The cell e.s.d.'s are taken into account individually in the estimation of e.s.d.'s in distances, angles and torsion angles; correlations between e.s.d.'s in cell parameters are only used when they are defined by crystal symmetry. An approximate (isotropic) treatment of cell e.s.d.'s is used for estimating e.s.d.'s involving l.s. planes.
Refinement. Refinement of *F*^2^ against ALL reflections. The weighted *R*-factor *wR* and goodness of fit *S* are based on *F*^2^, conventional *R*-factors *R* are based on *F*, with *F* set to zero for negative *F*^2^. The threshold expression of *F*^2^ > σ(*F*^2^) is used only for calculating *R*-factors(gt) *etc*. and is not relevant to the choice of reflections for refinement. *R*-factors based on *F*^2^ are statistically about twice as large as those based on *F*, and *R*- factors based on ALL data will be even larger.

### Fractional atomic coordinates and isotropic or equivalent isotropic displacement parameters (Å^2^)

**Table:**

	*x*	*y*	*z*	*U*_iso_*/*U*_eq_	
N1	0.32094 (13)	0.29578 (16)	0.92231 (7)	0.0578 (4)	
N2	0.34893 (13)	0.35212 (17)	0.97530 (7)	0.0597 (4)	
N3	0.16718 (12)	0.07857 (14)	0.00747 (7)	0.0541 (4)	
N4	0.17677 (13)	0.16075 (15)	0.05364 (7)	0.0563 (4)	
O1	0.17630 (13)	0.27945 (15)	0.83520 (7)	0.0765 (5)	
H1	0.2056	0.3111	0.8652	0.115*	
O2	0.21621 (14)	0.50002 (16)	0.95829 (7)	0.0969 (6)	
O3	0.47037 (12)	0.36530 (14)	1.07416 (6)	0.0750 (4)	
O4	0.23773 (13)	−0.12009 (15)	−0.03935 (7)	0.0826 (5)	
H4	0.2346	−0.0665	−0.0137	0.124*	
O5	0.31539 (14)	0.04667 (16)	0.10021 (7)	0.0888 (5)	
O6	0.11005 (11)	0.36046 (13)	0.10849 (6)	0.0684 (4)	
C1	0.33944 (15)	0.14134 (19)	0.85116 (8)	0.0549 (5)	
C2	0.24499 (17)	0.1865 (2)	0.81688 (9)	0.0602 (5)	
C3	0.2162 (2)	0.1340 (2)	0.76206 (10)	0.0767 (6)	
H3	0.1532	0.1645	0.7397	0.092*	
C4	0.2791 (2)	0.0384 (2)	0.74065 (10)	0.0797 (7)	
H4A	0.2583	0.0035	0.7041	0.096*	
C5	0.3730 (2)	−0.0064 (2)	0.77297 (10)	0.0780 (6)	
H5	0.4165	−0.0709	0.7581	0.094*	
C6	0.40240 (18)	0.0440 (2)	0.82710 (10)	0.0710 (6)	
H6	0.4662	0.0127	0.8486	0.085*	
C7	0.37168 (15)	0.1933 (2)	0.90977 (8)	0.0563 (5)	
C8	0.45543 (18)	0.1287 (2)	0.95166 (9)	0.0759 (6)	
H8A	0.5221	0.1802	0.9590	0.114*	
H8B	0.4789	0.0519	0.9344	0.114*	
H8C	0.4190	0.1126	0.9884	0.114*	
C9	0.29454 (16)	0.4577 (2)	0.98954 (9)	0.0606 (5)	
C10	0.33544 (15)	0.52031 (19)	1.04600 (8)	0.0569 (5)	
C11	0.41947 (15)	0.4758 (2)	1.08692 (8)	0.0577 (5)	
C12	0.44709 (18)	0.5422 (2)	1.13798 (9)	0.0729 (6)	
H12	0.5020	0.5115	1.1654	0.087*	
C13	0.3940 (2)	0.6530 (3)	1.14833 (11)	0.0827 (7)	
H13	0.4127	0.6967	1.1829	0.099*	
C14	0.3136 (2)	0.6999 (2)	1.10817 (12)	0.0865 (7)	
H14	0.2788	0.7759	1.1150	0.104*	
C15	0.28485 (19)	0.6335 (2)	1.05760 (11)	0.0733 (6)	
H15	0.2300	0.6655	1.0305	0.088*	
C16	0.56308 (19)	0.3193 (3)	1.11158 (10)	0.0882 (8)	
H16A	0.6237	0.3799	1.1146	0.132*	
H16B	0.5926	0.2444	1.0950	0.132*	
H16C	0.5353	0.3026	1.1504	0.132*	
C17	0.08689 (14)	0.00942 (18)	−0.08365 (8)	0.0505 (5)	
C18	0.15800 (15)	−0.09596 (19)	−0.08333 (9)	0.0607 (5)	
C19	0.14807 (18)	−0.1806 (2)	−0.12944 (11)	0.0790 (7)	
H19	0.1947	−0.2508	−0.1284	0.095*	
C20	0.07172 (17)	−0.1636 (2)	−0.17628 (10)	0.0748 (6)	
H20	0.0673	−0.2208	−0.2071	0.090*	
C21	0.00145 (18)	−0.0615 (2)	−0.17773 (10)	0.0701 (6)	
H21	−0.0511	−0.0493	−0.2095	0.084*	
C22	0.00875 (16)	0.0225 (2)	−0.13226 (9)	0.0627 (5)	
H22	−0.0401	0.0909	−0.1337	0.075*	
C23	0.09365 (15)	0.10144 (17)	−0.03562 (8)	0.0516 (5)	
C24	0.01927 (17)	0.2152 (2)	−0.03747 (10)	0.0704 (6)	
H24A	−0.0360	0.2105	−0.0069	0.106*	
H24B	−0.0208	0.2209	−0.0755	0.106*	
H24C	0.0670	0.2871	−0.0313	0.106*	
C25	0.25190 (16)	0.1365 (2)	0.09986 (9)	0.0572 (5)	
C26	0.25187 (15)	0.22604 (17)	0.15067 (8)	0.0524 (5)	
C27	0.18211 (15)	0.33129 (18)	0.15560 (9)	0.0552 (5)	
C28	0.18812 (18)	0.4015 (2)	0.20709 (10)	0.0680 (6)	
H28	0.1409	0.4706	0.2105	0.082*	
C29	0.2635 (2)	0.3694 (2)	0.25311 (10)	0.0747 (6)	
H29	0.2664	0.4168	0.2876	0.090*	
C30	0.33427 (19)	0.2686 (2)	0.24897 (10)	0.0744 (6)	
H30	0.3862	0.2483	0.2800	0.089*	
C31	0.32742 (17)	0.1977 (2)	0.19816 (9)	0.0640 (6)	
H31	0.3749	0.1286	0.1956	0.077*	
C32	0.0333 (2)	0.4636 (2)	0.11268 (11)	0.0884 (7)	
H32A	−0.0163	0.4512	0.1453	0.133*	
H32B	−0.0129	0.4708	0.0765	0.133*	
H32C	0.0776	0.5379	0.1190	0.133*	
H2	0.4078 (13)	0.330 (2)	1.0002 (8)	0.080*	
H4B	0.1314 (15)	0.2279 (14)	0.0561 (9)	0.080*	

### Atomic displacement parameters (Å^2^)

**Table:**

	*U*^11^	*U*^22^	*U*^33^	*U*^12^	*U*^13^	*U*^23^
N1	0.0546 (9)	0.0627 (11)	0.0555 (10)	0.0014 (8)	−0.0037 (8)	0.0059 (8)
N2	0.0567 (10)	0.0669 (11)	0.0546 (10)	0.0094 (9)	−0.0078 (8)	0.0061 (9)
N3	0.0520 (9)	0.0543 (10)	0.0559 (10)	0.0033 (8)	0.0014 (8)	0.0039 (8)
N4	0.0548 (9)	0.0540 (10)	0.0599 (10)	0.0068 (8)	−0.0006 (8)	0.0027 (9)
O1	0.0811 (10)	0.0791 (11)	0.0680 (10)	0.0200 (9)	−0.0146 (8)	0.0047 (8)
O2	0.0924 (11)	0.0879 (12)	0.1065 (12)	0.0312 (10)	−0.0459 (10)	−0.0091 (10)
O3	0.0696 (9)	0.0887 (11)	0.0652 (9)	0.0255 (8)	−0.0161 (7)	0.0016 (8)
O4	0.0723 (9)	0.0714 (10)	0.1018 (12)	0.0242 (8)	−0.0265 (9)	−0.0125 (9)
O5	0.0913 (11)	0.0879 (11)	0.0855 (11)	0.0431 (10)	−0.0196 (9)	−0.0160 (9)
O6	0.0639 (8)	0.0594 (9)	0.0810 (10)	0.0151 (7)	−0.0093 (7)	0.0014 (8)
C1	0.0500 (10)	0.0588 (12)	0.0562 (11)	−0.0027 (9)	0.0049 (9)	0.0117 (10)
C2	0.0605 (12)	0.0613 (13)	0.0590 (13)	0.0002 (11)	0.0038 (10)	0.0109 (10)
C3	0.0808 (15)	0.0853 (17)	0.0629 (14)	0.0030 (14)	−0.0124 (12)	0.0035 (13)
C4	0.0867 (16)	0.0848 (17)	0.0674 (14)	−0.0089 (14)	−0.0005 (13)	−0.0085 (13)
C5	0.0758 (15)	0.0801 (16)	0.0784 (15)	0.0034 (13)	0.0076 (13)	−0.0091 (13)
C6	0.0620 (12)	0.0771 (16)	0.0738 (15)	0.0055 (12)	0.0013 (11)	0.0023 (13)
C7	0.0484 (10)	0.0649 (13)	0.0557 (12)	0.0010 (10)	0.0036 (9)	0.0140 (10)
C8	0.0722 (14)	0.0903 (17)	0.0645 (13)	0.0219 (13)	−0.0069 (11)	0.0079 (12)
C9	0.0521 (11)	0.0626 (13)	0.0665 (13)	0.0036 (10)	−0.0057 (10)	0.0109 (11)
C10	0.0504 (10)	0.0592 (13)	0.0611 (12)	−0.0015 (10)	0.0018 (9)	0.0094 (10)
C11	0.0492 (11)	0.0680 (14)	0.0560 (12)	−0.0017 (10)	0.0051 (9)	0.0073 (10)
C12	0.0639 (13)	0.0939 (18)	0.0607 (13)	−0.0043 (13)	−0.0005 (10)	0.0002 (13)
C13	0.0853 (16)	0.0885 (19)	0.0746 (16)	−0.0078 (15)	0.0056 (13)	−0.0140 (14)
C14	0.0950 (18)	0.0719 (16)	0.0931 (18)	0.0080 (14)	0.0084 (15)	−0.0084 (15)
C15	0.0737 (14)	0.0649 (15)	0.0810 (16)	0.0070 (12)	−0.0010 (12)	0.0020 (12)
C16	0.0708 (14)	0.109 (2)	0.0834 (16)	0.0260 (14)	−0.0206 (12)	0.0132 (15)
C17	0.0426 (9)	0.0536 (11)	0.0557 (11)	−0.0009 (9)	0.0060 (8)	0.0085 (9)
C18	0.0443 (10)	0.0618 (13)	0.0758 (14)	0.0031 (10)	−0.0020 (10)	0.0009 (11)
C19	0.0604 (13)	0.0698 (15)	0.1064 (18)	0.0114 (12)	−0.0043 (13)	−0.0211 (14)
C20	0.0588 (13)	0.0823 (17)	0.0833 (16)	−0.0042 (12)	0.0015 (12)	−0.0186 (13)
C21	0.0653 (13)	0.0808 (16)	0.0640 (13)	−0.0014 (12)	−0.0014 (10)	0.0029 (12)
C22	0.0615 (12)	0.0649 (13)	0.0615 (12)	0.0060 (11)	0.0022 (10)	0.0097 (11)
C23	0.0459 (10)	0.0524 (11)	0.0569 (11)	0.0018 (9)	0.0082 (9)	0.0099 (9)
C24	0.0713 (13)	0.0676 (14)	0.0717 (14)	0.0174 (11)	−0.0035 (11)	0.0031 (11)
C25	0.0501 (11)	0.0600 (13)	0.0614 (12)	0.0036 (10)	−0.0003 (9)	0.0036 (10)
C26	0.0461 (10)	0.0505 (11)	0.0605 (12)	−0.0033 (9)	0.0023 (9)	0.0054 (10)
C27	0.0477 (10)	0.0529 (12)	0.0647 (12)	−0.0042 (9)	0.0003 (9)	0.0055 (10)
C28	0.0673 (13)	0.0553 (13)	0.0815 (15)	−0.0005 (11)	0.0033 (12)	−0.0061 (12)
C29	0.0824 (15)	0.0703 (15)	0.0708 (15)	−0.0098 (13)	−0.0035 (12)	−0.0101 (12)
C30	0.0778 (14)	0.0761 (16)	0.0679 (14)	−0.0004 (13)	−0.0138 (11)	0.0008 (13)
C31	0.0610 (12)	0.0641 (13)	0.0663 (13)	0.0025 (10)	−0.0038 (10)	0.0064 (11)
C32	0.0823 (15)	0.0685 (15)	0.113 (2)	0.0279 (13)	−0.0103 (14)	0.0000 (14)

### Geometric parameters (Å, °)

**Table:**

N1—C7	1.290 (2)	C12—H12	0.9300
N1—N2	1.368 (2)	C13—C14	1.369 (3)
N2—C9	1.347 (3)	C13—H13	0.9300
N2—H2	0.896 (9)	C14—C15	1.377 (3)
N3—C23	1.289 (2)	C14—H14	0.9300
N3—N4	1.370 (2)	C15—H15	0.9300
N4—C25	1.356 (2)	C16—H16A	0.9600
N4—H4B	0.899 (9)	C16—H16B	0.9600
O1—C2	1.355 (2)	C16—H16C	0.9600
O1—H1	0.8200	C17—C22	1.400 (2)
O2—C9	1.215 (2)	C17—C18	1.405 (3)
O3—C11	1.367 (2)	C17—C23	1.470 (3)
O3—C16	1.427 (2)	C18—C19	1.386 (3)
O4—C18	1.352 (2)	C19—C20	1.362 (3)
O4—H4	0.8200	C19—H19	0.9300
O5—C25	1.217 (2)	C20—C21	1.370 (3)
O6—C27	1.361 (2)	C20—H20	0.9300
O6—C32	1.430 (2)	C21—C22	1.369 (3)
C1—C2	1.401 (3)	C21—H21	0.9300
C1—C6	1.401 (3)	C22—H22	0.9300
C1—C7	1.470 (3)	C23—C24	1.500 (3)
C2—C3	1.388 (3)	C24—H24A	0.9600
C3—C4	1.363 (3)	C24—H24B	0.9600
C3—H3	0.9300	C24—H24C	0.9600
C4—C5	1.372 (3)	C25—C26	1.499 (3)
C4—H4A	0.9300	C26—C31	1.389 (3)
C5—C6	1.367 (3)	C26—C27	1.401 (3)
C5—H5	0.9300	C27—C28	1.386 (3)
C6—H6	0.9300	C28—C29	1.373 (3)
C7—C8	1.498 (3)	C28—H28	0.9300
C8—H8A	0.9600	C29—C30	1.367 (3)
C8—H8B	0.9600	C29—H29	0.9300
C8—H8C	0.9600	C30—C31	1.378 (3)
C9—C10	1.502 (3)	C30—H30	0.9300
C10—C15	1.386 (3)	C31—H31	0.9300
C10—C11	1.399 (3)	C32—H32A	0.9600
C11—C12	1.383 (3)	C32—H32B	0.9600
C12—C13	1.370 (3)	C32—H32C	0.9600
			
C7—N1—N2	118.64 (16)	O3—C16—H16A	109.5
C9—N2—N1	119.21 (16)	O3—C16—H16B	109.5
C9—N2—H2	115.1 (14)	H16A—C16—H16B	109.5
N1—N2—H2	125.2 (14)	O3—C16—H16C	109.5
C23—N3—N4	118.66 (15)	H16A—C16—H16C	109.5
C25—N4—N3	119.30 (16)	H16B—C16—H16C	109.5
C25—N4—H4B	117.8 (14)	C22—C17—C18	116.45 (18)
N3—N4—H4B	122.7 (14)	C22—C17—C23	121.50 (17)
C2—O1—H1	109.5	C18—C17—C23	122.05 (17)
C11—O3—C16	119.98 (17)	O4—C18—C19	117.16 (18)
C18—O4—H4	109.5	O4—C18—C17	122.82 (19)
C27—O6—C32	119.43 (17)	C19—C18—C17	120.02 (19)
C2—C1—C6	116.99 (19)	C20—C19—C18	121.6 (2)
C2—C1—C7	121.85 (18)	C20—C19—H19	119.2
C6—C1—C7	121.17 (18)	C18—C19—H19	119.2
O1—C2—C3	116.81 (19)	C19—C20—C21	119.6 (2)
O1—C2—C1	122.93 (19)	C19—C20—H20	120.2
C3—C2—C1	120.2 (2)	C21—C20—H20	120.2
C4—C3—C2	120.8 (2)	C22—C21—C20	119.8 (2)
C4—C3—H3	119.6	C22—C21—H21	120.1
C2—C3—H3	119.6	C20—C21—H21	120.1
C3—C4—C5	120.1 (2)	C21—C22—C17	122.6 (2)
C3—C4—H4A	119.9	C21—C22—H22	118.7
C5—C4—H4A	119.9	C17—C22—H22	118.7
C6—C5—C4	119.8 (2)	N3—C23—C17	116.08 (16)
C6—C5—H5	120.1	N3—C23—C24	122.58 (18)
C4—C5—H5	120.1	C17—C23—C24	121.34 (16)
C5—C6—C1	122.0 (2)	C23—C24—H24A	109.5
C5—C6—H6	119.0	C23—C24—H24B	109.5
C1—C6—H6	119.0	H24A—C24—H24B	109.5
N1—C7—C1	115.04 (17)	C23—C24—H24C	109.5
N1—C7—C8	123.38 (19)	H24A—C24—H24C	109.5
C1—C7—C8	121.56 (19)	H24B—C24—H24C	109.5
C7—C8—H8A	109.5	O5—C25—N4	121.80 (19)
C7—C8—H8B	109.5	O5—C25—C26	121.87 (17)
H8A—C8—H8B	109.5	N4—C25—C26	116.32 (17)
C7—C8—H8C	109.5	C31—C26—C27	117.67 (18)
H8A—C8—H8C	109.5	C31—C26—C25	115.32 (17)
H8B—C8—H8C	109.5	C27—C26—C25	126.98 (17)
O2—C9—N2	121.67 (19)	O6—C27—C28	122.40 (18)
O2—C9—C10	121.6 (2)	O6—C27—C26	117.57 (18)
N2—C9—C10	116.69 (17)	C28—C27—C26	120.03 (18)
C15—C10—C11	117.8 (2)	C29—C28—C27	120.3 (2)
C15—C10—C9	115.91 (18)	C29—C28—H28	119.9
C11—C10—C9	126.25 (19)	C27—C28—H28	119.9
O3—C11—C12	122.77 (18)	C30—C29—C28	120.9 (2)
O3—C11—C10	117.10 (18)	C30—C29—H29	119.6
C12—C11—C10	120.1 (2)	C28—C29—H29	119.6
C13—C12—C11	120.3 (2)	C29—C30—C31	119.0 (2)
C13—C12—H12	119.8	C29—C30—H30	120.5
C11—C12—H12	119.8	C31—C30—H30	120.5
C14—C13—C12	120.6 (2)	C30—C31—C26	122.1 (2)
C14—C13—H13	119.7	C30—C31—H31	118.9
C12—C13—H13	119.7	C26—C31—H31	118.9
C13—C14—C15	119.3 (2)	O6—C32—H32A	109.5
C13—C14—H14	120.3	O6—C32—H32B	109.5
C15—C14—H14	120.3	H32A—C32—H32B	109.5
C14—C15—C10	121.8 (2)	O6—C32—H32C	109.5
C14—C15—H15	119.1	H32A—C32—H32C	109.5
C10—C15—H15	119.1	H32B—C32—H32C	109.5

### Hydrogen-bond geometry (Å, °)

**Table:**

*D*—H···*A*	*D*—H	H···*A*	*D*···*A*	*D*—H···*A*
O1—H1···N1	0.82	1.82	2.535 (2)	144
O4—H4···N3	0.82	1.82	2.541 (2)	145
N2—H2···O3	0.90 (1)	1.83 (2)	2.591 (2)	141 (2)
N4—H4B···O6	0.90 (1)	1.88 (2)	2.619 (2)	138 (2)
